# Harnessing the Therapeutic Potential of Extracellular Vesicles for Oral Wound Healing

**DOI:** 10.3390/bioengineering13020148

**Published:** 2026-01-27

**Authors:** Helly A. Patel, Bianca Schmiliver, Keerthi Priya Chinniyampalayam Sekar, Mirelle Dogini, Chidubem Onyeagoro, Daniel C. Shah, M. Hope Robinson, Babatunde Giwa-Otusajo, David T. Wu, Steven L. Goudy

**Affiliations:** 1 Division of Otolaryngology, Department of Pediatrics, Emory University School of Medicine, Atlanta, GA 30322, USAbiancaschmil@gmail.com (B.S.);; 2 The Wallace H. Coulter Department of Biomedical Engineering, Georgia Tech and Emory University, Atlanta, GA 30322, USAdshah314@gatech.edu (D.C.S.);; 3 Department of Oral Medicine, Infection, and Immunity, Harvard School of Dental Medicine, Boston, MA 02115, USA; 4 Harvard John A. Paulson School of Engineering and Applied Sciences, Cambridge, MA 02139, USA; 5 Wyss Institute for Biologically Inspired Engineering, Harvard University, Boston, MA 02115, USA; 6 Division of Periodontics, Faculty of Dental Medicine and Oral Health Sciences, Montreal, QC H3A 0C7, Canada; 7 Children’s Healthcare of Atlanta, Atlanta, GA 30329, USA

**Keywords:** oral wound healing, extracellular vesicles, mucosal healing, cell-free regenerative therapies

## Abstract

Oral wound healing is a robust process; however, complications from surgery, systemic diseases, and aging can impair healing. While some treatments exist, regenerative therapies to promote mucosal wound healing remain limited. In recent years, there has been a significant rise in FDA-approved cell-based therapies; however, extracellular vesicles represent an emerging cell-free alternative that may mitigate risks associated with cellular therapies, including tumorigenesis and immunogenicity. These lipid-encapsulated nanovesicles can deliver therapeutic cargo, such as proteins, lipids, nucleic acids, or drugs, to the wound site. Extracellular vesicles can be derived from mesenchymal stromal cells, immune cells, bodily fluids, or bacteria, and engineered through genetic modification, preconditioning, or direct cargo loading to enhance therapeutic potency. Furthermore, advanced delivery platforms, including hydrogels, microneedles, and aerosols, allow for sustained and localized EV delivery to the oral wound site. This review examines differences between cutaneous and oral wound healing; factors that impair oral repair; extracellular vesicle sources and engineering strategies; and delivery strategies for developing EV-based therapeutics for oral wound healing.

## 1. Introduction

**Figure 1:**
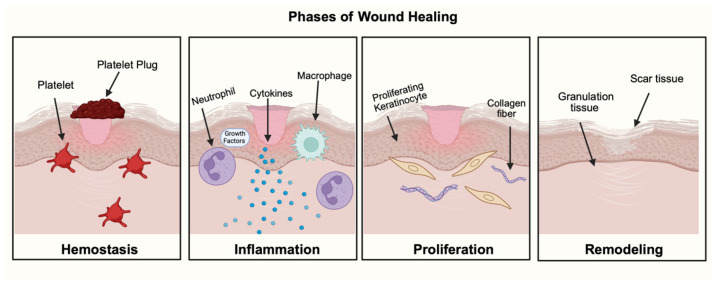
Phases of Wound Healing.

Cutaneous and oral wound healing share fundamental overlapping phases, including hemostasis, inflammation, proliferation, and remodeling, which can be visualized by Figure 1 [1,2]. Upon injury, hemostasis halts bleeding by constricting the blood vessel and forming a platelet plug [3]. Immune cells such as neutrophils then arrive at the wound site and phagocytize bacteria and debris [4]. Monocytes are recruited and mature into macrophages to further orchestrate healing [5]. By releasing growth factors and cytokines, immune cells can facilitate the transition to the proliferative phase [6]. During the proliferative phase, keratinocytes and fibroblasts migrate to the wound site to allow for tissue re-epithelialization [7,8]. Lastly, during the remodeling phase, granulation tissue forms, which then eventually matures into a scar [9]. An important distinction of oral wounds is the tendency to heal faster with reduced scarring compared to cutaneous wounds [10]. These advantages arise due to unique environmental factors, including salivary histatins, increased tissue vascularity, and oral microbiota that can positively influence the wound-healing cascade [11,12,13,14,15]. Additionally, various subtypes of oral stem cells, progenitor cells, and fibroblast populations contribute to these advantages. For example, a study performed by Ko et al. found that the increased population of postnatal paired-related homeobox-1+ (Prx1+) fibroblast cells was found to increase oral mucosal healing compared to the mice that lacked those cells [16]. Despite these inherent advantages, oral wound healing can be associated with prolonged healing, extreme pain, and complications due to surgery, trauma, infection, aging, or systemic diseases [17,18,19].

Oral wounds are a significant global burden with substantial associated mortality and morbidity rates. Severe conditions such as noma can carry mortality rates up to 90% without treatment, while oral mucositis affects up to 30% to 40% of patients on chemotherapy [20]. Beyond mortality, the morbidity burden is also considerable. One study found 87.6% of participants to have one or more oral mucosal lesions, and approximately one billion people have experienced a traumatic dental injury before [21,22]. Wound healing complications following oral surgery or trauma can lead to significant disability and poor clinical prognosis. For example, following cleft lip and palate surgery, the formation of an oronasal fistula (ONF) is associated with nasal regurgitation and hypernasal speech, necessitating multiple revision surgeries that may be unsuccessful [23].

Regenerative medicine therapies are promising approaches that can be used synergistically to promote oral wound healing. Current therapies include platelet-rich plasma isolated from the patient’s own blood, which can be used to promote postoperative tissue healing [24]. Additionally, the Orasoothe “Sockit” gel is a hydrogel wound dressing that promotes oral wound healing and is used after dental procedures, such as tooth extractions, to prevent dry socket [25]. However, most regenerative therapies for the oral cavity remain in early stages of development. Recent literature suggests that extracellular vesicles (EVs) are emerging regenerative cell-free therapeutics that can be applicable for wound healing, as summarized in Table 1; however, the application of EVs to oral wound healing remains largely underexplored [26,27]. This review paper highlights key differences between oral and cutaneous wound-healing processes, appropriate sources of EVs for oral wounds, methods to engineer EVs, and practical EV-delivery methods.

## 2. Mechanistic Differences Between Oral and Cutaneous Wound Healing

To engineer extracellular vesicle therapeutics for oral wounds, it is essential to consider the mechanistic differences between cutaneous and oral wounds. The oral cavity is continuously bathed in saliva, a bioactive fluid rich in growth factors, antimicrobial peptides, and extracellular vesicles [49,50,51,52]. However, this wet nature of the oral cavity makes the application of therapies difficult due to poor adhesion compared to cutaneous wounds [53]. Histologically, the skin and oral mucosa appear structurally similar, both lined by stratified squamous epithelium; however, the oral mucosa contains basal lamina, lamina propria, and the submucosa, while cutaneous skin is made up of the dermis and hypodermis [54,55,56,57]. Furthermore, the oral epithelium is thicker and characterized by a higher basal proliferation rate, creating an abundance of keratinocytes for re-epithelialization [10,58]. The underlying lamina propria in the oral cavity is also more vascularized than the dermis, ensuring a prompt influx of oxygen, nutrients, and immune cells after injury [59,60]. By contrast, the cutaneous skin’s less dense vascular network requires the formation of new vessels to meet the metabolic demands of repair [60].

At the cellular and molecular level, oral wounds follow a distinct trajectory. Genetically, the oral cavity is primed with activated repair genes such as sex-determining region Y-box 2 (*SOX2*) and paired-like homeodomain 1 (*PITX*) before injury, whereas in the skin, these genes are turned on following injury. These transcriptional regulators were found to increase cell migration and enhance oral wound resolution in vivo [61]. Another study identified 181 transcription factors as being basally upregulated in oral keratinocytes compared with immortalized cutaneous skin [62]. Furthermore, cellular subtypes such as STAT3-activated SPRR1B+ keratinocytes are present in unwounded mucosa but absent in skin, which suggests their role in priming the oral mucosa [63]. Thus, the oral mucosa is primed by specific cellular subtypes and genes that contribute to its intrinsic capacity for rapid healing.

The inflammatory response following oral injury is rapid and robust but resolves quickly, marked by an efficient transition from pro-inflammatory (M1-like) macrophages to anti-inflammatory and pro-reparative (M2-like) macrophages [6,64,65,66]. This rapid resolution of the inflammatory phase of wound healing is critical for limiting fibrosis and priming the wound-healing microenvironment for tissue remodeling [67,68]. The skin, by contrast, has a longer presence of immune cells that contribute to a pro-fibrotic environment [10,69]. Furthermore, crosstalk between the oral microbiome and immune cells can influence the progression of inflammation, leading to a pathological, chronic inflammatory wound state [70]. In the context of wound healing, commensal bacteria were found to elicit an M2-like phenotype, whereas pathogenic oral bacteria, such as *Porphyromonas gingivalis* (*P. gingivalis*), elicit M1-like inflammatory mediators [71]. Fibroblast phenotypes further amplify the differences between oral and cutaneous wound healing, with oral fibroblasts expressing lower levels of alpha-smooth muscle actin (alpha-SMA), producing less pro-fibrotic cytokine transforming growth factor beta 1 (TGFB1), and a balanced production of matrix-remodeling enzymes [58,72,73,74]. Cutaneous dermal fibroblasts, on the other hand, readily differentiate into myofibroblasts under TGFB1 signaling, driving wound contraction and the deposition of dense, parallel collagen fibers that characterize scarring [75]. Together, these inflammatory and stromal differences between cutaneous and oral wound healing help explain why oral wounds heal faster, mount a robust immune response, and exhibit reduced fibrosis compared with cutaneous wounds [10].

## 3. Clinical Complications of Poor Oral Wound Healing

While the oral mucosa is recognized for its remarkable scarless healing capacity, this advantage can be compromised. Local, systemic, lifestyle, and therapeutic factors may interfere with the tightly coordinated processes of oral mucosal repair [76,77]. When these factors interfere with oral wound healing, oral wounds may close more slowly, remain inflamed for an extended period, and undergo fibrotic changes, ultimately increasing the risk of persistent, non-healing wounds [78].

### 3.1. Local Factors

The oral environment itself can contribute to healing, as outlined in Table 2. When the oral microbiome is balanced, it can reduce inflammation and promote wound healing [79]. When this balance is disrupted, pathogenic species can dominate, leading to a state known as dysbiosis [80]. Pathogenic bacteria, such as *Porphyromonas gingivalis* (*P. gingivalis*), release virulence factors, including lipopolysaccharides, and can activate inflammasomes, including the NACHT, LRR [leucine-rich repeat] and PYD [pyrin domain] domains-containing protein 3 (NLRP3) inflammasome [81,82]. The NLRP3 inflammasome mediates caspase-1 activation and promotes the secretion of proinflammatory cytokines, thereby exacerbating inflammation [83].

Common oral pathogenic bacteria include *P. gingivalis, Enterococcus faecalis* (*E. Faecalis*), and *Streptococcus mutans* (*S. mutans*). Furthermore, in periodontal disease, the red complex is a specific group of bacteria that includes *Treponema denticola* (*T. denticola*), *Porphyromonas gingivalis*, and *Tannerella forsythia* (*T. forsythia*) and is known to have an increased association with periodontitis [93].

Extracellular vesicles secreted by these pathogenic bacteria facilitate the inflammatory response associated with dysbiosis. One study found that oral epithelial cells infected with *P. gingivalis* significantly increased the production of EVs that carry tumor necrosis factor alpha (TNF-a) and interleukin 1 beta (IL-1B) [86]. Another study found that *E. Faecalis* EVs promoted M1 polarization of macrophages through the NOD2/RIPK2 signaling pathway, which is involved in the response to bacterial infections and the induction of an inflammatory response [94]. *S. mutans* plays a key role in cavity development, and its EVs contain virulence proteins that can contribute to biofilm formation and disease progression [88]. Other pathogenic bacteria, such as *Fusobacterium nucleatum*, stimulate the secretion of interleukin-6 (IL-6), interleukin-8 (IL-8), and tumor necrosis factor alpha (TNF-a) [95,96].

However, emerging evidence suggests that probiotics, such as *Lactobacillus reuteri* (*L. reuteri*) or its metabolite reuterin, may help restore microbial balance, support stem cell activity, and accelerate repair [79]. A study found that *L. reuteri* membrane vesicles reduced pro-inflammatory factors such as TNF-a, IL1B, and IL-6, increased the number of CD206+ macrophages, and upregulated M2-like macrophage production [89]. *Lactobacillus acidophilus* (*L. acidophilus*) and *Lactobacillus plantarum* probiotic strains have also been studied for their wound healing properties by Gudadappanavar et al., who found that *L. acidophilus* significantly enhanced wound healing by promoting contraction and accelerating epithelialization [97].

Saliva also plays a pivotal role in wound healing [49]. Due to the abundance of growth factors, histatins, and antimicrobial peptides, saliva can stimulate keratinocyte migration and protect against infection [11,90]. When salivary flow is reduced, for example, as a result of radiotherapy, systemic disease, autoimmune conditions such as Sjögren’s syndrome, or medication use, these protective effects are decreased [98,99]. Patients who developed oral mucositis due to radiation therapy were found to have lower salivary epidermal growth factor (EGF), which reduces the ability of the oral mucosal wound healing [100,101]. Additionally, salivary dysfunction can further delay wound closure in diabetic patients [92,102]. Clearly, the importance of local factors for healing in the oral environment can be better understood in the context of the entire system.

### 3.2. Systemic Conditions and Comorbidities

Systemic diseases play a decisive role in derailing oral wound healing. In patients with diabetes, persistent hyperglycemia drives the accumulation of advanced glycation end-products and reactive oxygen species (ROS), both of which interfere with angiogenesis and prolong inflammation [103,104,105]. Clinically, poorly controlled diabetes is associated with xerostomia, candidiasis, periodontal disease, and delayed postsurgical repair [106,107,108,109,110]. This relationship is bidirectional: chronic oral inflammation can exacerbate insulin resistance and destabilize glycemic control, thereby further impairing wound healing [111]. To expand, it was found that after intensive periodontal treatment, hemoglobin A1c (HbA1c) levels were found to be 0.6% lower than those of patients who had the control periodontal treatment [112]. This further builds upon the bidirectional relationship that proper dental care can improve glycemic control and influence oral wound healing.

States of immunosuppression, including HIV infection, organ transplantation, and chemotherapy, can disrupt the oral immune balance needed for repair [113,114,115]. Furthermore, EV expression is altered in immunosuppressed patients and varies by disease type [116].

### 3.3. Iatrogenic and Therapeutic Factors of Impaired Oral Wound Healing

Iatrogenic factors that negatively influence the process of oral wound healing frequently emerge as a consequence of clinical interventions. During oral surgeries, trauma caused by excessive tissue manipulation and improper suturing techniques can disrupt the delicate balance of cellular and extracellular matrix interactions essential for tissue repair [117]. Additionally, the use of certain local anesthetics with vasoconstrictors may reduce blood flow, further complicating healing by limiting the delivery of essential nutrients and oxygen to the affected area [118]. These surgical occurrences can lead to negative outcomes such as chronic wounds attributable to infections and tissue loss resulting from necrosis.

Clinical interventions and therapeutic agents can also significantly impair oral wound healing. Radiotherapy and chemoradiation, widely used in head and neck cancer treatment, are particularly detrimental. Beyond the acute effects of mucositis and salivary gland dysfunction, radiation induces accumulation of DNA damage and reactive oxygen species in epithelial, endothelial, and stromal cells, leading to apoptosis and senescence [119,120,121]. At the immune level, one study showed that mouse RAW 264.7 macrophage cells exposed to radiation increased the expression of triggering receptor expressed on myeloid cells 1 (Trem1), which exacerbates nuclear factor kappa B (NF-kB)-mediated inflammatory responses and changes macrophages toward an M1-like phenotype [122]. Another study found that ionizing radiation significantly downregulated anti-inflammatory genes such as cluster of differentiation 163 (*CD163*), mannose receptor C-type 1 (*MRC1*), and versican (*VCAN*), while upregulating cluster of differentiation 80 (*CD80*), in macrophages, further promoting a pro-inflammatory environment [123].

Agents that target bone remodeling create additional challenges. At a mechanistic level, prolonged suppression of osteoclast-mediated bone resorption results in accumulation of microdamage and impaired coupling between osteoclasts and osteoblasts, limiting the bone’s ability to remodel in response to mechanical and inflammatory stresses, which can develop into a persistent oral mucosal wound with devascularized bone underneath [124,125,126,127]. Once established, the combination of necrotic bone, chronic infection, and reduced vascular supply creates a chronic cycle of inflammation and non-healing.

Long-term use of corticosteroids and nonsteroidal anti-inflammatory drugs (NSAIDs) can also blunt the inflammatory cascades required to initiate repair [128]. Corticosteroids inhibit NF-kB signaling and reduce the production of key pro-inflammatory cytokines and chemokines, thereby reducing neutrophil and macrophage recruitment and activation in the early wound phase [129]. Collectively, these iatrogenic factors create an oral environment less responsive to injury and more prone to chronic complications.

### 3.4. Patient Related/Modifiable Risk Factors

Lifestyle behaviors are essential modifiers of wound healing. Exposure of the oral mucosa to smoking restricts blood flow through vasoconstriction, reduces oxygen delivery, and interferes with fibroblast activity [130,131]. More recent work has shown that smoking reprograms macrophages toward an M1-like phenotype. Amerio et al. found that smokers have a significantly higher number of M1-like macrophages in the oral mucosa compared to non-smokers with peri-implantitis lesions, which could potentially contribute to tissue destruction and poor wound healing [132].

Cellular senescence further amplifies the risk of poor oral wound healing due to impaired angiogenesis, reduced fibroblast proliferation, and collagen turnover decline [133]. The presence of senescent cells, which have ceased cell cycle progression, reduces the turnover and proliferation of healthy cells [134]. Furthermore, senescent cells secrete molecules of the senescent-associated secretory phenotype (SASP), which can exacerbate inflammation [135]. Cell cycle inhibitors such as p21 inhibit cell cycle progression and induce senescence [136]. In a study by Gasek et al., clearing cells with high p21 expression accelerated wound closure by partially inhibiting NF-kB, a cytokine implicated in inflammation and in maintaining a M1-like macrophage phenotype [137]. The presence of multiple chronic conditions and the use of numerous medications, both common in older adults, can further exacerbate vulnerabilities to oral wound healing, contributing to slower and less predictable healing outcomes.

### 3.5. Introduction to Extracellular Vesicles

**Figure 2:**
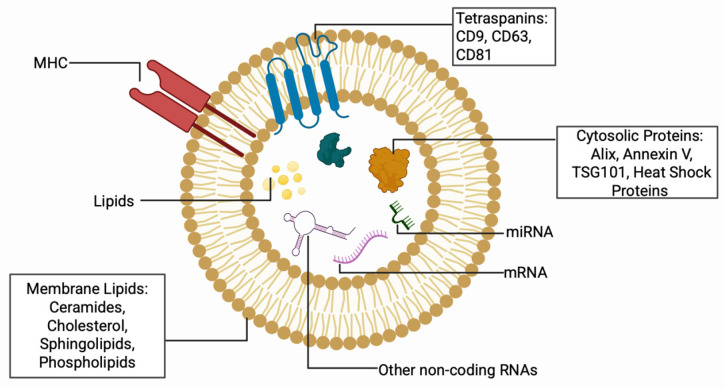
Extracellular Vesicle Composition.

Cell-based therapies have shown promise for tissue repair; however, the risks of tumorigenicity and immunogenicity have limited their clinical translation [138,139,140]. Extracellular vesicles offer a cell-free alternative that preserves therapeutic benefits while mitigating these complications [141]. Extracellular vesicles are nanoscale lipid-encapsulated vesicles that carry various cargoes, including DNA, RNA, bioactive lipids, and proteins, that play a crucial role in intercellular communication, and their composition is illustrated in Figure 2 [142].

These vesicles are produced through distinct biogenesis pathways, with exosomes originating from multivesicular endosomes and microvesicles budding directly from the plasma membrane [143,144]. However, given the rise in extracellular vesicle-based studies, the International Society of Extracellular Vesicles (ISEV) has established specific guidelines to ensure uniformity and reproducibility [145]. Furthermore, the Minimal Information for Studies of Extracellular Vesicles (MISEV) 2023 guidelines recommend the adoption of the umbrella term “extracellular vesicles” until the biogenesis-specific terminology like “exosomes” and “ectosomes” has been confirmed. Furthermore, markers such as CD63, CD9, and CD81 or endosomal pathway proteins such as tumor susceptibility gene 101 (TSG101), programmed cell death 6 interacting protein (PDCD6IP), often referred to as ALIX, and Glyceraldehyde 3-phosphate dehydrogenase (GAPDH) can be used to characterize EVs [145,146]. This shift reduces classification ambiguity in applications by making it easier to differentiate subpopulations generated by current isolation methods. Extracellular vesicles can further be classified based on their diameter into subpopulations such as small EVs (50–150 nm), medium EVs (200–800 nm), and large EVs (>1000 nm) [147].

**Figure 3:**
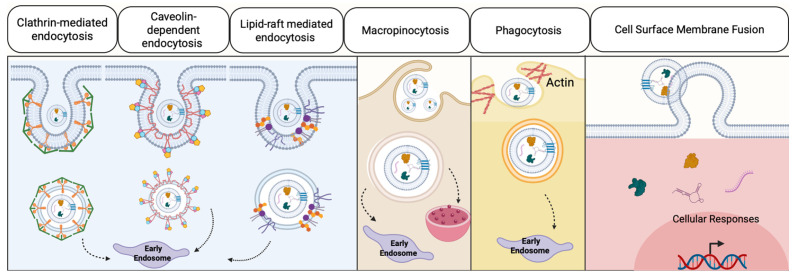
Mechanisms for Extracellular Vesicle Uptake.

Extracellular vesicles deliver their therapeutic cargo through multiple mechanisms, as visualized in Figure 3. One method is through endocytosis, which can include caveolin-mediated uptake, clathrin-dependent pathway, micropinocytosis, phagocytosis, and lipid raft-mediated internalization [148,149,150]. Beyond endocytosis, they can also fuse with the membrane and become internalized [151]. Selective cargo sorting during EV biogenesis can enhance therapeutic potency beyond that of parental cells, by potentially concentrating pro-wound healing molecules within the vesicles [152,153].

Extracellular vesicles demonstrate versatility in both diagnostic and therapeutic applications [154]. Diagnostically, their cargo composition, comprising proteins, nucleic acids, and lipids, reflects the physiological state of the parent cells, enabling disease detection [155]. Therapeutically, EVs can function as a delivery vehicle that can be sourced from various cell types and bodily fluids, depending on the intended application [156]. This adaptability, combined with their reduced immunogenicity and absence of replication risk, positions extracellular vesicles as a next-generation therapeutic platform that can overcome many of the limitations of traditional cell therapies.

In the case of wound healing, extracellular vesicles are promising therapeutics that can deliver bioactive cargo to regulate differentiation, promote adhesion, and stimulate cell proliferation [27,157,158,159]. By modulating recipient cell behavior, extracellular vesicles could influence each phase of the wound healing process.

### 3.6. Harvesting Extracellular Vesicles

**Figure 4:**
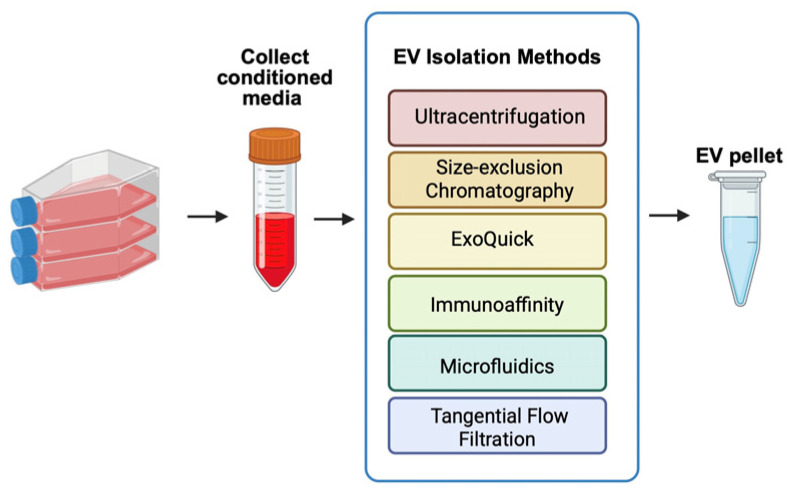
Extracellular Vesicle Isolation Methods.

Extracellular vesicles can be harvested from conditioned cell culture media or biological fluids through many isolation techniques, as illustrated in Figure 4. The most commonly employed methods include ultracentrifugation, size-exclusion chromatography, precipitation-based approaches, immunoaffinity capture, microfluidics, and tangential flow filtration [160,161].

Ultracentrifugation remains the gold standard for isolating components, involving sequential centrifugation steps at progressively higher speeds [162]. This method can isolate EVs with average sizes of 122, 89, and 60 nm [163]. While this method can achieve high particle yields, it can decrease EV purity. Size-exclusion chromatography is quicker than ultracentrifugation, but yields fewer total particles [164]. Precipitation-based approaches, such as ExoQuick, reduce EV solubility and allow for isolation at low-speed centrifugation. However, this method can also yield lower purity due to protein co-precipitation [165]. Immunoaffinity targeting of surface markers on EVs can increase specificity and purity for specific EV subpopulations [166]. Microfluidics can use a very small amount of liquid and can achieve both high-yield and high-purity EVs [167]. Lastly, tangential flow filtration also yields high EV yield and purity by allowing fluid to flow parallel to the membrane surface, thereby concentrating EVs [164].

### 3.7. Potential Sources of Extracellular Vesicles for Oral Wound Healing

The selection of extracellular vesicle sources for oral wound healing involves considering both the inherent therapeutic properties derived from parental cells and the compatibility of these vesicles with the oral microenvironment. Given the unique environment of the oral cavity, EVs from mammalian and non-mammalian sources can be used for wound healing, as the diverse array of potential sources, ranging from various mammalian and non-mammalian cell sources and probiotic bacteria to milk and saliva, can provide either inherent therapeutic benefits or serve as engineerable carriers for targeted wound healing interventions [168,169,170]. Current applications of extracellular vesicles in oral conditions are summarized in Table 3.

### 3.8. Mesenchymal Stromal Cells

Mesenchymal stromal cells (MSCs) are a popular choice for wound healing research and have been extensively reviewed by numerous studies due to their immunomodulatory properties [178,179,180]. Additionally, their scalability and FDA approval make them an attractive candidate for clinical use [181,182]. These multipotent stem cells can be derived from various sources, including bone marrow, adipose tissue, the umbilical cord, and dental tissues such as dental pulp and periodontal ligament [183]. Functional differences may also be present depending on the MSC source. MSC-derived EVs delivered in preclinical wound healing models demonstrate wound-healing capabilities in cutaneous tissues, including the promotion of angiogenesis, cellular proliferation, and anti-inflammatory responses, which are crucial for wound repair [183,184,185,186,187]. However, the role of EVs derived from MSCs in treating oral wounds has been the subject of limited study.

In a study performed by Sun et al., exosomes derived from human gingival mesenchymal stromal cells were found to suppress the inflammatory response by regulating the expression of NF-kB signaling and WNT5A in LPS-induced periodontal ligament stem cells (PDLSCs) and inhibiting the overexpression of *NFKB* and *IL1B* mRNA that was upregulated after the lipopolysaccharide (LPS) induction in vitro [34].

Additionally, Gao et al. used extracellular vesicles from human umbilical cord mesenchymal stem cells to coat oral mucositis in rats and also found that topical application of umbilical cord MSC EVs to oral wounds promoted healing by also inhibiting the activation of the NF-kB signaling pathway [38].

Furthermore, miRNA hsa-let-7e-5p was found to be highly upregulated in human umbilical cord MSC extracellular vesicles and to alleviate oral mucositis by repressing TGF-beta activated kinase 1 (MAP3K7) binding protein 2 (TAB2) expression [171]. The TAB2 adapter protein is involved in the formation of the kinase complex that activates NF-kB [188]. Thus, MSC-derived extracellular vesicles may promote oral wound healing by regulating the NF-kB inflammatory signaling pathway.

Lastly, Zhang et al. found that xeno-free induced gingival MSCs conditioned media contained an increased number of EVs that attenuated macrophage inflammation [189].

Prolonged inflammation can lead to delayed oral wound healing, resulting in the formation of chronic oral wounds. The immunomodulatory properties of MSC-derived extracellular vesicles could potentially attenuate the persistent inflammatory cascade and help the transition to the proliferative and remodeling phase.

Guo et al. irradiated mice with injured salivary glands and injected adipose-derived stem cell EVs and found the mice to have a 96% increase in saliva secretion. The EV treatment regulated epithelia-mesenchymal transition through the TGFβ1/Smad3 pathway, potentially through miR-199a-3p [173].

### 3.9. Macrophage

Macrophages contribute to debris clearance and modulation of the inflammatory milieu. Through phenotypic transitions from M1-like (pro-inflammatory) to M2-like (anti-inflammatory) states, these cells can resolve inflammation by secreting anti-inflammatory cytokines such as IL-10, arginase (ARG), and TGF-b, along with growth factors that promote cellular proliferation and angiogenesis [190,191,192]. When delivering a miR-30e-5p inhibitor in the macrophage extracellular vesicles, it prevented periodontitis in mice with an ovariectomy [174].

M2-like macrophage-derived extracellular vesicles have demonstrated efficacy in promoting angiogenesis. Lyu et al. demonstrated that M2-like macrophage-derived exosomes enhance angiogenic response in cutaneous wound healing models [193]. Building on this finding, subsequent work revealed that these macrophage-derived EVs improve skin flap survival through activation of the HIF1AN/HIF-1a/VEGFA signaling axis, further amplifying neovascularization [194]. Such findings suggest potential applications in preventing oral wound complications following major surgical procedures, including oral flaps utilized in cleft palate reconstruction.

Beyond angiogenesis, M2-like macrophage-derived extracellular vesicles can attenuate excessive inflammatory responses. In a diabetic cutaneous wound model, M2-derived exosomes promoted macrophage polarization toward the M2 phenotype while inhibiting uncontrolled inflammation [195]. Although the precise mechanisms remain incompletely understood, one study identified exosomal miR-590-3p as a mediator of reduced inflammation and enhanced epithelial regeneration through activation of the YAP/β-catenin pathway in an ulcerative colitis model [196]. This mechanism may have translational relevance to oral wound healing, given the structural and functional similarities between colonic and oral mucosal tissues.

### 3.10. Fibroblasts

Fibroblasts migrate to the wound site, where they deposit extracellular matrix (ECM) components that provide the structural scaffold for tissue regeneration [197]. Fibroblast-derived extracellular vesicles (FEVs) have been found to enhance multiple aspects of wound healing, including cellular proliferation, migration, angiogenesis, and scar reduction in vitro [48]. The therapeutic potential of FEVs has been further demonstrated through their delivery in a gelatin methacrylate (GelMA) hydrogel system, which accelerated cutaneous wound closure in a murine model [198]. Another study found that human dermal fibroblast-derived EVs were internalized by keratinocytes and fibroblasts, increased cell proliferation and migration, and reduced early inflammatory responses after a dermal excisional injury [199]. One study applied the human gingival fibroblast secretome to a murine excisional wound and found increased re-epithelialization, reduced inflammation, and increased angiogenesis [200]. Thus, the extracellular vesicles found within the secretome could be further studied to determine their role in this process. Additionally, gingival fibroblasts are currently being studied for scarless cutaneous wound healing applications; their EVs hold promise for intraoral regenerative therapies given their tissue-specific origin and inherent compatibility with the oral mucosal environment [201,202].

### 3.11. Saliva

Salivary extracellular vesicles have been primarily investigated for their diagnostic applications; however, emerging evidence supports their therapeutic potential in wound healing [203]. Epithelial cells and granulocytes in the oral cavity can secrete EVs into saliva [204]. The parotid and submandibular glands secrete pure glandular saliva, and the cellular origin of the extracellular vesicle depends on the cells that make up the secreting gland [204,205]. Saliva itself has demonstrated the capacity to stimulate oral and skin wound closure in vitro [11], due to the presence of histatins, small peptides found in saliva that confer wound healing and antimicrobial properties [206].

Recent advances in organoid technology have allowed for a more controlled investigation of salivary gland-derived EVs. Qian et al. created a human minor salivary gland acinar-like organoid that secretes exosomes capable of promoting cellular proliferation and angiogenesis while delivering growth factors to the wound site. Proteomic analysis revealed that these organoid-derived exosomes are enriched in proteins such as vinculin (VCL), collagen type I alpha 1 chain (COL1A1), collagen type II alpha 1 chain (COL2A), integrin subunit beta 1 (ITGB1), and mitogen-activated protein kinase kinase 2 (MAP2K2), to name a few, which are involved in wound repair, immune modulation, and coagulation, thereby suggesting they recapitulate key reparative functions of native saliva in a more targeted manner [207].

Salivary exosomes have been found to promote human umbilical vein endothelial cell (HUVEC) proliferation, migration, and tube formation in vitro, with corresponding acceleration of cutaneous wound closure in vivo. Mechanistically, these EVs are enriched in mRNA encoding the ubiquitin conjugating enzyme E2O (*UBE2O*), which promotes angiogenesis by degrading SMAD family member 6 (SMAD6), and activating bone morphogenetic protein 2 (BMP2) signaling pathways critical for neovascularization [208]. Beyond angiogenic effects, salivary exosomes also modulate wound healing through metabolic reprogramming of fibroblasts. Building on these findings, salivary exosomes have been shown to promote wound closure through metabolic reprogramming of fibroblasts, whereby exosome-associated glycolytic enzymes enhance glycolytic flux, leading to increased matrix metallopeptidase 1 (MMP1) and matrix metallopeptidase 3 (MMP3) secretion and accelerated extracellular matrix (ECM) remodeling [209]. Clearly, saliva plays a multifactorial role in oral wound healing, with a reduction in saliva from radiation or systemic disease (e.g., Sjogrens Syndrome) associated with poor wound healing and EVs generated from saliva may be key to improving poor wound healing [210,211].

### 3.12. Milk

Bovine milk is an abundant and readily accessible source of extracellular vesicles with demonstrated innate wound healing properties or drug delivery potential. A specific subpopulation of milk extracellular vesicles endogenously expresses Connexin 43 (Cx43), a gap junction protein implicated in intercellular communication during tissue repair [212]. Functionally, bovine milk extracellular vesicles promote fibroblast proliferation and migration, while colostrum-derived EVs are particularly enriched in cytokines such as the anti-inflammatory Interleukin-4 (IL-4), chemokine (C-X-C motif) ligand 10 (CXCL10) which is involved in tissue remodeling and leukemia inhibitory factor (LIF) which is expressed in response to keratin damage, that facilitate the transition from inflammatory to proliferative phases of wound healing [169]. These bovine EVs have been shown to enhance re-epithelialization, stimulate angiogenesis, and promote extracellular matrix maturation, thereby making them an attractive, cost-effective, and scalable therapeutic agent for wound management [213,214].

### 3.13. Bacteria

Bacterial extracellular vesicles (BEVs) can be taken up by surrounding bacteria to restore microbial homeostasis, ultimately influencing wound healing. When Chen et al. induced a circular tongue ulcer in mice and used *Lactobacillus reuteri* EVs, these EVs were taken up by macrophages, which then subsequently shifted their phenotype toward M2 [89].

Notably, BEVs derived from oral commensal bacteria demonstrate species-specific regenerative properties. For example, *Streptococcus mutans*-derived EVs enhance epithelial proliferation through a toll-like receptor 3 (TLR3)-dependent mechanism mediated by a transfer RNA methionine (tRNA-Met) variant in an oral mucosal organoid [175]. Similarly, EVs isolated from *Lactobacillus rhamnosus* GG, a probiotic strain, accelerated wound closure through the miR-21-5p-mediated signaling pathway [170]. These findings suggest that specific bacterial EVs could serve as microbiome-modulating therapeutics to address oral wound-healing complications associated with dysbiosis.

### 3.14. Platelet

Platelet-derived EVs (PEVs) isolated from platelet lysate enhance oral wound healing in both gingival fibroblasts and keratinocytes. Antich-Rosselló et al. demonstrated that PEVs accelerated wound closure in vitro and modulated gene expression related to extracellular matrix remodeling [176].

### 3.15. Dental Pulp Stem Cell

Dental pulp stem cell-derived EVs (DPSC-EVs) are ideal as they are accessed from oral tissue. Qiao et al. showed that DPSC-EVs promoted proliferation, migration, and osteogenic differentiation of periodontal ligament stem cells and modulated the inflammatory microenvironment by inhibiting the IL-6/JAK2/STAT3 signaling pathway. Additionally, the DPSC-EVs polarized macrophages to an M2-like phenotype and in a rat periodontitis model, the EV treatment reduced alveolar bone loss and promoted healing of the periodontal epithelium [177].

## 4. Engineering Extracellular Vesicles for Oral Wound Healing

In order to optimize EV-based therapeutics, various engineering strategies can be implemented to modify the genetic material or signaling molecules that make up the cargo or to modify surface properties to enhance homing to the tissue site or cellular uptake. EV engineering can occur prior to isolation with modifications to the parental cell or after, by manipulating the EV pellet directly. This section will further elaborate on current modification strategies.

### 4.1. Genetic Engineering of Parental Cells

Due to their innate ability to deliver genetic material to cells, viral vectors are commonly used in gene-editing applications [215]. Lentiviral vectors are derived from HIV-1 and can be used in the laboratory to stably integrate genetic material. This benefit of stable genetic modification is important in EV engineering for loading, in this case, proteins or RNA cargo relevant to oral wound repair [216]. These systems are particularly valuable for loading EVs with growth factors (VEGF, PDGF, FGF2), anti-inflammatory miRNAs, and antimicrobial peptides, which can be delivered to the wound site to promote healing. Vascular endothelial growth factor (VEGF) promotes wound healing by facilitating angiogenesis [217]. Platelet-derived growth factor (PDGF) facilitates fibroblast recruitment, cellular proliferation, and migration [218]. Fibroblast growth factor 2 (FGF2) plays many roles, but has been found to stimulate wound healing [219].

Adenoviral vectors offer high transduction efficiency for transient modifications, while adeno-associated virus (AAV) systems enable tissue-specific targeting through the selection of specific serotypes [220]. For example, human umbilical cord mesenchymal stem cells were infected with miR-150-5p using lentiviral vectors, and their isolated extracellular vesicles were found to stably express miR-150-50 and promote wound healing by activating the phosphatidylinositol 3-kinase (PI3K)/AKT pathway through phosphatase with tensin homology (PTEN). This pathway activation and enhanced protection against H_2_O_2_-injured human keratinocyte cells and promoted the growth and migration of these cells while inhibiting apoptosis [221].

### 4.2. Pharmacological and Environmental Preconditioning

Preconditioning parental cells by altering their growth or by treatment with drugs known to alter cell cargo can also be used to engineer pro-regenerative EVs. Hypoxic preconditioning is when cells are exposed to low oxygen conditions, and in this environment, hypoxia can increase the secretion of growth factors such as VEGF and can stimulate wound healing by activating hypoxia inducible factor-1 (HIF-1) [222]. This is usually downregulated in chronic conditions such as diabetes, so isolating out EVs that have been engineered in these conditions could be therapeutically advantageous for diabetic wound healing purposes [223].

Parental cells can also be treated with drugs that alter cargo. Preconditioning of MSCs with thrombin resulted in increased EV production, and these EVs were enriched with VEGF and angiogenin [224]. Importantly, application of these VEGF and angiogenin-enriched EVs leads to cutaneous wound healing in vivo [225]. Lastly, Shah et al. found that MSCs exogenously treated with sphingomyelinase, an enzyme that hydrolyzes sphingomyelin into ceramide, were found to alter EV cargo, specifically enriching pro-wound healing and pro-angiogenic proteins and miRNAs [226]. Furthermore, the generation of EV-liposome hybrids could enhance drug loading into the vesicle [227,228].

### 4.3. Passive EV Loading

**Figure 5:**
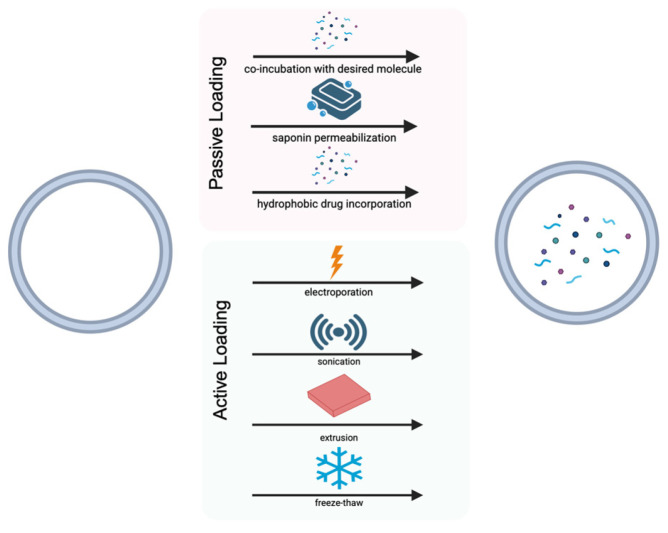
Active and Passive Extracellular Vesicle Engineering Strategies.

Passive loading methods do not require the addition of energy to deliver cargo into the vesicle. Specific passive loading methods include co-incubation, saponin permeabilization, and hydrophobic drug incorporation, as visualized in Figure 5. Hydrophobic drug incorporation involves applying the principle of diffusion through a concentration gradient, which allows the entry of desired lipophilic molecules into the vesicle without disrupting the lipid bilayer membrane [229]. Saponin is a detergent used to gently permeabilize membranes and allow molecules to enter vesicles [230]. EVs derived from epidermal stem cells were loaded with VH298 (VH-EVs), a von Hippel-Lindau inhibitor, by co-incubation at varying concentrations. These VH-EVs were then found to promote diabetic cutaneous wound healing by promoting angiogenesis [231].

### 4.4. Active EV Loading

Active loading methods work by transiently permeating the lipid membrane of the EV, thereby facilitating the desired cargo into the vesicle. Standard active loading practices include electroporation, sonication, extrusion, and freeze–thaw according to Figure 5. Electroporation uses an electric pulse that can create pores in the membrane, allowing cargo to be trapped within the vesicle once the membrane is resealed [232]. Sonication utilizes ultrasound waves to disrupt the lipid bilayer, enabling cargo to enter. While sonication has high loading efficiency, the particle yield is significantly decreased. On the other hand, electroporation allows for a higher yield but at a lower efficiency [233]. The extrusion method allows EVs to move through a membrane that has a pore size that is similar to their homogenous size, while simultaneously allowing for drugs to enter the vesicle [234]. Lastly, freeze–thaw involves cycles of freezing and thawing to permeate the membrane, enabling it to create ice crystals with pores that incorporate drugs within the vesicle. The membrane then fuses again, encapsulating the drugs [235,236].

### 4.5. Cell Surface Engineering

Beyond cargo alteration, the EV membrane itself can also be engineered. Methods such as genetic modification, click chemistry, lipid insertion, metabolic labeling, affinity binding, and enzymatic ligation can be used to alter the membrane [237]. Furthermore, membrane-bound moieties can improve EV stability and enable targeted delivery. For example, one study found that EVs engineered with polyethylene glycol (PEG) remained in plasma for more than an hour [238]. Another study chemically conjugated a collagen-binding peptide, SILY (SILY-EVs), which binds collagen. The SILY-EVs were delivered to a mouse hind-limb ischemia model and improved in situ retention, dampened inflammatory responses, and enhanced vascularization [239].

### 4.6. Engineering Challenges

There are some engineering challenges to consider when selecting an appropriate loading methodology. First, precise control over drug encapsulation efficiency remains limited, resulting in variable loading capacities that can compromise reproducibility [240]. Furthermore, scaling up engineered EV therapeutics can pose substantial economic barriers due to the cost of complex production protocols [241]. The physicochemical properties of therapeutic cargo significantly influence loading feasibility [242]. Larger RNA molecules, such as mRNA and plasmids, can be difficult to deliver within the vesicle due to their negative charge and large size. Growth factors and proteins require preservation of tertiary and quaternary structures that can be impeded by the physical and chemical stress of active loading methods.

### 4.7. Advances in EV Delivery Strategies

**Figure 6:**
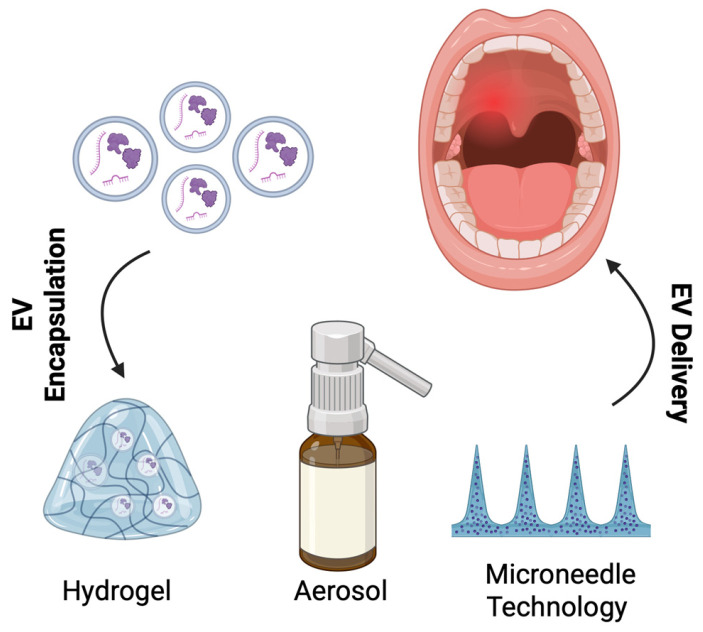
Extracellular Vesicle Delivery Methods for the Oral Cavity.

Many delivery methods can be optimized to deliver EVs to the oral wound site, which can be visualized in Figure 6.

### 4.8. Hydrogels

Hydrogels are three-dimensional polymer networks that retain substantial amounts of water, which supports their structural integrity [243]. Combined with their tunable mechanical properties, hydrogels can mimic the native extracellular matrix and serve as potential biocompatible applications for soft tissue engineering [244]. When extracellular vesicles are encapsulated within hydrogels, the biodegradable nature, by extension, can allow for sustained extracellular vesicle release. The degradation kinetics can be further tailored to respond to specific pH conditions, allowing for timely extracellular vesicle release. For example, a study conducted by Chewchuk et al. demonstrated that extracellular vesicles encapsulated in a hydrogel containing the CD9 binding protein enabled the controlled release of EVs in response to acidic environments found in damaged tissues [245]. Furthermore, due to their amorphous nature, extracellular vesicles encapsulated within the hydrogel can be either topically applied or injected into the wound site, simultaneously protecting vesicles from enzymatic degradation and other clearance mechanisms within the mouth. In terms of application in the oral cavity, the adhesive properties of hydrogels make them an appropriate extracellular vesicle delivery method.

Hydrogel groups vary due to their polymer source, crosslinking method, ionic charge, and their behavior in various conditions [246]. The source for hydrogels can be natural, synthetic, or a combination of the two. Natural polymers include polysaccharides (cellulose, chitosan, alginate, and hyaluronic acid) and proteins (collagen, gelatin, and fibrin) [247]. Synthetic polymers include poly(ethylene) glycol (PEG), polylactic acid (PLA), and polycaprolactone (PCL) [248]. However, chitosan-based hydrogels have been most commonly studied in EV research. The natural polymer can maintain pH stability and allow for sustained EV release [249]. One study found that the delivery of a chitosan/silk hydrogel containing encapsulated gingival mesenchymal stem cell-derived extracellular vesicles in vivo increased neo-epithelium and collagen production [250]. Gel MA derived from gelatin loaded with EVs derived from keratinocytes enhanced wound healing in diabetic mouse skin ulcers by promoting vascularization and also found that the GelMA-EVs were released continuously for 14 days [250].

### 4.9. Microneedle Technology

An alternate non-invasive technique compared to localized EV injection is a delivery system that uses microneedle patches to deliver EVs to the target site. Furthermore, these microneedles allow for long-term storage for EVs. To deliver EVs into damaged tissues, one study developed a hydrogel microneedle patch that was loaded with stem cell-derived mitochondria-rich EVs which they found to promote macrophage polarization toward the M2-like phenotype and effectively sustain the delivery of these EVs. The application of a sustained delivery EV microneedle patch could be of use in chronic oral wounds [251]. Moreover, another study used anti-microbial hydrogel microneedle patches loaded with adipose tissue-derived apoptotic vesicles that were able to allow the wound to heal in a scarless manner and inhibit the bacteria in the infected wound [252]. This idea could be used in the context of oral wound healing because a disrupted oral microbiome can sustain chronic inflammation and hinder the formation of healthy oral tissue.

### 4.10. Aerosols

Aerosols are another optimal platform for extracellular vesicle delivery to the mouth. Spraying extracellular vesicles onto the wound site could be a non-invasive way of delivering EVs to the wound site. He et al. developed a hydrogel spray composed of methacrylate-modified oxidative hyaluronic acid (OHAMA) and poly-e-L-lysine (EPL), loaded with small extracellular vesicles derived from stem cells, which achieved a 92.63% skin wound closure rate after treatment [253]. Furthermore, aerosols have been positively accepted by patients in terms of adherence [254]. However, it is important to consider the potential risks associated with inhalation of the aerosol.

### 4.11. Challenges and Opportunities

While extracellular vesicles are a promising therapeutic approach to enhance oral wound healing, several barriers must be overcome to enable clinical translation. EV production can result in heterogeneous batches, low yield, or impure samples [255,256,257]. EV scalability remains a primary challenge, as current isolation methods, such as ultracentrifugation, are time-consuming, and consistent, cost-effective, high-volume manufacturing remains a key challenge. As noted previously, the MISEV guidelines aim to standardize across the field; however, diverse isolation protocols can yield heterogeneous EV populations. Their short half-life also makes it difficult to sustain therapeutic benefits. More specifically, within six hours, EVs were found to be eliminated from circulation via hepatic and renal routes [258]. Furthermore, current storage methods can decrease EV efficacy over time, with freeze–thaw cycles diminishing membrane integrity and cargo [259].

In the oral environment, parameters such as saliva flow, mechanical forces, and pH fluctuations can further complicate the efficacy of EVs. Lastly, limited pre-clinical research has studied EVs specifically for oral wound healing. However, there are significant benefits and opportunities for EV therapeutics for oral wound healing. For example, after surgery, EVs can be implanted or delivered within a hydrogel to increase healing. EVs derived from immune cells can modulate inflammation, reduce oral pain, and facilitate healing [260].

Several cell-based therapeutics are used to repair tissue. For example, Epicel uses autologous keratinocyte grafts to treat burns, while StrataGraft uses allogenic human keratinocytes and fibroblasts [261,262]. Clear FDA guidelines remain unavailable due to the challenges of large-scale extracellular vesicle manufacturing. However, with the recent FDA approval of the mesenchymal stem cell therapy Ryoncil, strides towards a homogenic MSC EV population could be made [182].

After isolation, EVs may still exhibit variation due to differences in the growth conditions of the cell source [263]. Areas of research to bring EVs to the market include scalable and reproducible EV isolation, improving purity, optimizing EV yield, and understanding the EV mechanism of action [264]. Despite challenges, ongoing investigations are underway to improve the clinical translation of EV therapeutics. For example, lyophilization is an emerging method that improves the long-term stability of extracellular vesicles, allowing for room-temperature storage [265].

## 5. Conclusions

In summary, extracellular vesicles are a promising cell-free therapeutic for oral wound healing applications. These lipid membrane-encapsulated vesicles can carry various cargoes that can be utilized for both therapeutic and diagnostic purposes. Furthermore, EVs can be isolated from various sources, ranging from mammalian cells and bodily fluids such as saliva to bacteria. This ability can be strategically optimized to facilitate uptake by desired target populations.

During biogenesis, EVs can concentrate cellular cargo or can be directly engineered to carry desired drugs or cargo. Extracellular vesicles are also promising therapeutics in personalized medicine, as they can be obtained directly from patients’ biological fluids or cells.

Many of the preclinical studies reviewed in this paper highlight their potential for application in oral models. However, more studies are needed to address limitations and facilitate clinical translation; furthermore, there are few studies demonstrating EV potential in oral wound-healing models. By adapting advancements from other study models, we can develop innovative methods to accelerate oral wound healing.

## Figures and Tables

**Table 1:** Role of Extracellular Vesicles During the Phases of Wound Healing.

First Author & Year	Phases	Key Cellular Events	Role of EVs	Molecular Pathway
Setua 2022 [28] Owens 2011 [29] Berckmans 2001 [30] Tao 2017 [31] Aatonen 2012 [32]	Hemostasis	Coagulation and initiation of repair	EVs derived from platelet and endothelial cells promote coagulation by providing procoagulant phospholipid surfaces and tissue factor activity.	Enhance thrombin generation and fibrin formation via tissue factor and phosphatidylserine exposure. Promote procoagulant activities and thrombin generation time by facilitating assembly of tenase and prothrombinase complexes.
Ding 2023 [33] Sun 2022 [34] Li 2016 [35] Peng 2024 [36] Shen 2021 [37]	Inflammation	Immune cell recruitment, cytokine modulation, and inflammation resolution	EVs derived from immune and stromal modulate cytokine release and promote macrophage M1 to M2 transition.	MSC and oral tissue-derived EVs regulate inflammatory cytokines and the NF-κB activity. Downregulate TLR4 pathway, TNF-α, IL-1β and elevate IL-10 and TGF-β to resolve inflammation.
Gao 2022 [38] Shahsavandi 2025 [39] Li 2025 [40] Ju 2023 [41] Zeng 2021 [42] Li 2025 [43]	Proliferation	Epithelial migration, fibroblast activation, angiogenesis/vasculogenesis	EVs transfer miRNAs and growth factors that enhance keratinocyte migration, angiogenesis, and fibroblast proliferation.	MSC-, epithelial, and fibroblast-derived EVs promote angiogenesis via VEGF pathway, PTEN/PI3K/AKT signaling, and NF-kB/MAPK pathway inhibition. Promote fibroblast migration through miR-21, PTEN/AKT pathway.
Yuan 2021 [44] Nawaz 2018 [45] Wang 2017 [46] Ahmadpour 2023 [47] Oh 2021 [48]	Remodeling	Extracellular matrix reorganization, scar reduction, tissue regeneration	EVs coordinate fibroblast to myofibroblast differentiation and accelerate wound closure via extracellular matrix and collagen remodeling.	EV miRNAs regulate TGF-β and Smad signaling leading to reduced fibrosis and scarring. EVs are active carriers of matrix-degrading enzymes (MMPS, ADAMs, cathepsins) and regulate extracellular matrix remodeling.

**Table 2:** Local Factors in the Oral Cavity.

Factors	Normal Function	Pathology	Mechanism of Impaired Healing	Examples
Oral microbiome	Maintains epithelial turnover and primes immune responses for rapid repair [84,85]	Dysbiosis—imbalance favoring pathogenic species [80].	Pathogen release virulence factors (e.g., LPS) that activate inflammatory pathways such as NLRP3 inflammasome [81], caspase-1 activation, and proinflammatory cytokine secretion [83]. Aberrant NLRP3 activation disrupts stromal cell function, delays epithelial closure, prolongs inflammation, and increases infection susceptibility.	—
Pathogenic bacteria	Balanced microbial community supports healing.	Overgrowth of pathogens causes chronic inflammation and epithelial stress.	Chronic inflammation damages epithelial barrier and delays healing.	*P. gingivalis* induces EVs carrying TNF-α, IL-1β, and promotes inflammation [86]; *E. faecalis* EVs trigger M1 macrophage polarization via NOD2/RIPK2 pathway, sustaining inflammation [87]; *S. mutans* EVs contain virulence proteins that promote biofilm formation and disease progression [88].
Probiotic bacteria	Supports microbial balance and anti-inflammatory effects.	Loss or absence reduces regenerative capacity.	*L. reuteri* EVs decrease TNF-α, IL-1β, IL-6; increase CD206+ and M2 macrophages, promoting anti-inflammatory healing [79,89]. Restores microbial balance, enhances stem cell activity, and accelerates tissue repair.	—
Saliva	Contains growth factors, histatins, and antimicrobial peptides that stimulate keratinocyte migration and protect from infection [90].	Reduced salivary flow (e.g., radiotherapy, autoimmune disease, medication use).	Xerostomia leads to increased microbial colonization, slower re-epithelialization, and higher infection risk [91].	In diabetes, salivary dysfunction delays wound closure [92].

**Table 3:** Preclinical Evidence for Extracellular Vesicle Applications in Oral Wound Repair.

First Author & Year	EV Source	Oral Injury	In Vitro Oral Wound Model	In Vivo Oral Wound Model	Main Findings
Sun 2022 [34]	Human Gingival Mesenchymal Stem Cells	Periodontitis	LPS was used to exacerbate inflammation in periodontal ligament stem cells	—	The human gingival mesenchymal stem cell exosomes dampened the inflammatory response by decreasing the expression of NF-kB and Wnt5a.
Gao 2022 [38]	Human Umbilical Mesenchymal Stem Cells	Oral Mucositis	—	Wistar rats (male 6–8 weeks) were treated with glacial acetic acid in the inner mucosa of the lower lipEV	EV treatment group resulted in a reduced immunostaining intensity of NF-kB, IL-6, and TNF-a.
Lin 2024 [171]	Human Umbilical Mesenchymal Stem Cells	Oral Mucositis	LPS-induced human oral keratinocytes	Hamster model for Oral mucositis	Human umbilical cord mesenchymal stem cell extracellular vesicles reduced inflammation after human oral keratinocyte exposure to LPS. EV treatment reduced OM phenotype in vivo. Bioinformatic analysis showed the overexpression of has-let-7e-5p in EV.
Zhang 2025 [172]	Human Gingiva-Derived Mesenchymal Stem Cell	Tongue Defect	—	Rat Tongue Defect Model	iGMSC-derived secretome applied to the rat tongue defect wound promoted regeneration without fibrosis and shape deformity. Secretome had increased IL-10 and suppressed TNF-a expression following LPS stimulation in macrophages.
Guo 2025 [173]	Human Adipose-Derived Stem Cell	Salivary glands injured with 14 Gy	Irradiated submandibular gland epithelial C6 (SMG-CG) cells	Irradiated injured salivary glands C57BL/6 mice	Mice with exosome treatment increased saliva secretion, cell proliferation, and tissue repair genes. miR-199a-3p was increased in mice with exosome treatment and reduced epithelial to mesenchymal transition and inactivated the TGFB1-Smad3p.
Li 2022 [174]	Macrophage	Periodontitis	—	Mice that have undergone an ovariectomy surgery with an induction of periodontitis	Therapeutic delivery of miR-30e-5p inhibitor in macrophage extracellular vesicles can treat periodontitis in an estrogen deficiency model.
Chen 2024 [89]	*Lactobacillus reuteri*	Oral Mucosa Wound Healing	—	C57BL/6 mice received a circular ulcer on the tongue	*Lactobacillus reuteri* extracellular vesicles were uptaken by macrophages shifted their phenotype to M2 by regulating mitochondrial permeability and decreasing oxidative stress.
Oh 2025 [175]	*Streptococcus mutans*	Oral Mucosal Organoids	Human oral tissue including buccal mucosa, tongue, and mandible mucosa were used to create an organoid	—	*Streptococcus mutans*-derived extracellular vesicles promoted epithelial proliferation and wound healing through the TLR3 mechanism facilitated by tRNA-Met variant.
Antich-Rossello 2022 [176]	Platelet	—	Human gingival keratinocyte and fibroblast scratch assay	—	Platelet-derived extracellular vesicles increased wound closure in both gingival keratinocyte and fibroblasts.
Qiao 2023 [177]	Dental Pulp Stem Cell	Periodontitis	—	Rat Periodontitis Model	Dental Pulp Stem Cell exosomes prompted healing of the periodontal epithelium by potentially inhibiting the IL-6/JAK2/STAT3 pathway.

## Data Availability

No new data were created or analyzed in this study.

## Abbreviations

The following abbreviations are used in this manuscript:

AAV	Adeno-associated virus
Alpha-SMA	Alpha-smooth muscle actin
ARG	Arginase
BEV	Bacterial extracellular vesicle
BMP2	Bone morphogenetic protein 2
CD	Cluster of differentiation
COL1A1	Collagen type I alpha 1 chain
COL2A	Collagen type II alpha 1 chain
Cx43	Connexin 43
CXCL10	Chemokine (C-X-C motif) ligand 10
ECM	Extracellular matrix
EGF	Epidermal growth factor
EPL	Poly-e-L-lysine
EV	Extracellular vesicle
FEV	Fibroblast-derived extracellular vesicle
FGF2	Fibroblast growth factor 2
GelMA	Gelatin methacrylate
GAPDH	Glyceraldehyde-3-phosphate dehydrogenase
HbA1c	Hemoglobin A1c
HIF-1	Hypoxia-inducible factor 1
HIF1AN	Hypoxia-inducible factor 1 subunit alpha inhibitor
HUVEC	Human umbilical vein endothelial cell
IL-1β	Interleukin-1 beta
IL-4	Interleukin-4
IL-6	Interleukin-6
IL-8	Interleukin-8
IL-10	Interleukin-10
ISEV	International Society of Extracellular Vesicles
ITGB1	Integrin subunit beta 1
LIF	Leukemia inhibitory factor
LPS	Lipopolysaccharide
LRR	Leucine-rich repeat
MAP2K2	Mitogen-activated protein kinase kinase 2
MAP3K7	Mitogen-activated protein kinase kinase kinase 7
MISEV	Minimal Information for Studies of Extracellular Vesicles
MSC	Mesenchymal stromal cell
MRC1	Mannose receptor C-type 1
MMP1	Matrix metallopeptidase 1
MMP3	Matrix metallopeptidase 3
NF-κB	Nuclear factor kappa-light-chain-enhancer of activated B cells
NLRP3	NLR family pyrin domain containing 3
NSAID	Nonsteroidal anti-inflammatory drug
OHAMA	Oxidized methacrylate hyaluronic acid
ONF	Oronasal fistula
PCL	Polycaprolactone
PDCD6IP/ALIX	Programmed cell death 6 interacting protein
PDGF	Platelet-derived growth factor
PDLSC	Periodontal ligament stem cell
PEG	Polyethylene glycol
PI3K	Phosphatidylinositol 3-kinase
PITX	Paired-like homeodomain
PLA	Polylactic acid
Prx1+	Paired-related homeobox-1 positive (fibroblast subtype)
PTEN	Phosphatase and tensin homolog
PYD	Pyrin domain
ROS	Reactive oxygen species
SASP	Senescence-associated secretory phenotype
SMAD6	Mothers against decapentaplegic homolog 6
SOX2	Sex-determining region Y-box 2
SILY	Collagen-binding peptide motif
STAT3	Signal transducer and activator of transcription 3
TAB2	TGF-β activated kinase 1 binding protein 2
TGF-β1	Transforming growth factor beta 1
TLR3	Toll-like receptor 3
TNF-α	Tumor necrosis factor alpha
Trem1	Triggering Receptor expressed on myeloid cells-1
tRNA-Met	Transfer RNA methionine
TSG101	Tumor susceptibility gene 101
UBE2O	Ubiquitin-conjugating enzyme E2O
VEGF	Vascular endothelial growth factor
VCL	Vinculin
VCAN	Versican
VH-EV	EVs loaded with VH298
WNT5A	Wingless-related integration site 5A
YAP	Yes-associated protein
